# Cinnamaldehyde ameliorates STZ-induced diabetes through modulation of autophagic process in adipocyte and hepatic tissues on rats

**DOI:** 10.1038/s41598-024-60150-2

**Published:** 2024-05-02

**Authors:** Nesma A. Ghazal, Yara T. Agamia, Basant K. Meky, Nagwa M. Assem, Wafaa M. Abdel-Rehim, Sara A. Shaker

**Affiliations:** Department of Biochemistry, Medical Research Institute, Alexandria University, 165 El-Horreya Avenue, EL-Hadara, POB 21561, Alexandria, Egypt

**Keywords:** Biochemistry, Biotechnology, Molecular biology

## Abstract

Type 2 diabetes mellitus is a worldwide public health issue. In the globe, Egypt has the ninth-highest incidence of diabetes. Due to its crucial role in preserving cellular homeostasis, the autophagy process has drawn a lot of attention in recent years, Therefore, the purpose of this study was to evaluate the traditional medication metformin with the novel therapeutic effects of cinnamondehyde on adipocyte and hepatic autophagy in a model of high-fat diet/streptozotocin-diabetic rats. The study was conducted on 40 male albino rats, classified into 2 main groups, the control group and the diabetic group, which was subdivided into 4 subgroups (8 rats each): untreated diabetic rats, diabetic rats received oral cinnamaldehyde 40 mg/kg/day, diabetic rats received oral metformin 200 mg/kg/day and diabetic rats received a combination of both cinnamaldehyde and metformin daily for 4 weeks. The outcomes demonstrated that cinnamaldehyde enhanced the lipid profile and glucose homeostasis. Moreover, Cinnamaldehyde had the opposite effects on autophagy in both tissues; by altering the expression of genes that control autophagy, such as miRNA 30a and mammalian target of rapamycin (mTOR), it reduced autophagy in adipocytes and stimulated it in hepatic tissues. It may be inferred that by increasing the treatment efficacy of metformin and lowering its side effects, cinnamaldehyde could be utilized as an adjuvant therapy with metformin for the treatment of type 2 diabetes.

## Introduction

Diabetes mellitus (DM) is a long-term metabolic condition marked by persistent hyperglycemia. It could be due to a lack of insulin secretion, resistance to insulin's peripheral activities, or both^1^. Almost all global cases (96%) are type 2 diabetes (T2D); all 16 risk factors studied were associated with T2D. High body mass index (BMI) was the primary risk for T2D—accounting for 52.2% of T2D disability and mortality—followed by dietary risks, environmental/occupational risks, tobacco use, low physical activity, and alcohol use^2^. Egypt is one of the most diabetic-affected countries, with 8.2 million diabetic patients in 2017, and that number is anticipated to rise to 16.7 million by 2045^3^.

Some studies indicate that autophagy plays a significant role in the study of human diseases, particularly in glucose metabolism. Understanding the mechanism of autophagy is crucial in researching diabetes mellitus and its complications^4^. The autophagic machinery plays a critical role in the pathogenesis of T2DM and controls the normal pancreatic beta cell activity. Increased autophagy, on the other hand, protects insulin-target organs like the liver, skeletal muscle, and adipose tissue against oxidative damage^5^. Autophagy in adipose tissue has been studied about T2DM as an additional major target of insulin, and thus a link between autophagy and adipogenesis has been developed^6^. Through compromised β-cell function and the development of IR, altered autophagic activity has been linked to the advancement of obesity in T2DM^7^, which suggested that hepatic autophagy was reduced in the presence of IR. Additionally, Codogno and Meijer^8^ reported that autophagy induction can improve IR and impaired autophagy causes impaired insulin sensitivity in obese people.

Each step of autophagosome formation is firmly synchronized by proteins encoded by autophagy-related genes (Atgs)^9^. A mammalian target of rapamycin (mTOR) kinase-containing protein complexes, which is a critical inhibitor of autophagy, is one of the major regulators. As a result, there's a lot of interest in seeing if mTOR inhibition can be used as a diabetes treatment^10^. Beclin- 1 complex is required for vesicle formation and to promote autophagosome-lysosome fusion^11^. Atg12 and light chain 3 (LC3) are two ubiquitin-like conjugation kinds of machinery involved in autophagosome elongation and closure, LC3-I is the cytosolic isoform of the protein, which is attached to phosphatidylethanolamine via two ubiquitin-like processes. The E1-like enzyme Atg7 and the E2-like enzyme Atg3 catalyze these reactions, resulting in membrane-bound LC3-II. As a result, LC3-II development has been observed in cell and animal systems as a marker for the production of autophagosomes^12^.

Metformin is the first-line treatment for T2DM. It's a complicated medication with various action sites and molecular processes. Metformin diminishes glucose synthesis by acting directly or indirectly on the liver, and it increases glucose utilization via acting on the gut. Metformin inhibits the mitochondrial respiratory chain in the liver at the molecular level, resulting in increased insulin sensitivity (through effects on fat metabolism) and decreased production of gluconeogenic enzymes^13^.

Traditional T2DM pharmaceutical therapies have several drawbacks, including side effects and a high probability of secondary failure. Cinnamaldehyde has the formula C6H5CH=CHCHO and is an organic molecule. It is a significant bioactive component that lowers blood glucose levels in diabetic rats by enhancing glucose absorption, improving insulin sensitivity in adipose tissue, increasing glycogen synthesis in the liver, and having antioxidant properties^14^. It has antibacterial properties and is known to reduce the generation of inflammatory cytokines^15^. Although many randomized controlled trials (RCTs) have revealed the benefits of cinnamon on T2DM, the effects of cinnamon supplementation on glycemic control in patients with T2DM are inconclusive^16^. Therefore, the purpose of this study was to evaluate the traditional medication metformin with the novel therapeutic effects of cinnamaldehyde on adipocyte and hepatic autophagy in a model of high-fat diet/streptotocin-diabetic rats.

## Materials and methods

### Experimental animals

The study was conducted on 40 male albino rats 2 months old purchased from the animal house of Medical Research Institute, Alexandria, Egypt. All rats had free access to food and water with 12:12 hours of light/dark cycle and constant environmental conditions before experimentation and thereafter.

### Ethical statement

All experiments were pursued by the standards of the National Institutes of Health Guide for the Care and Use of Laboratory Animals (NIH Publications No. 8023, revised 1978) and were performed after the approval of the Institutional Animal Care and Use Committee (IACUC)-Alexandria University, Egypt (Approval No.: AU01220062911 and AU01219073023). The study also followed ARRIVE guidelines and complied with the National Research Council’s guide for the care and use of laboratory animals.

### Type 2 diabetes mellitus induction

Type 2 diabetes mellitus was induced according to the method of Srinivasan^17^. Rats were fed an in-house prepared high-fat diet (HFD) for 4 weeks.

The HFD consisted of commercial rat chow plus peanuts, milk chocolate, and sweet biscuits in a proportion of 3:2:2:1. All components of the high-fat diet were ground and blended^18^. The HFD-fed rats exhibited significantly high plasma glucose, insulin, triglycerides, and total cholesterol levels as compared to normally-fed control rats. After 4 weeks of dietary manipulation, the rats were injected intraperitoneally (i.p.) with a low dose of Streptozotocin (STZ) (Sigma Chemicals, USA) (55 mg/kg b.wt.) dissolved in citrate buffer (pH 4.4). After 2 days of STZ injection, the rats with fasting blood glucose levels of ≥ 200 mg/dl measured by Glucometer (ACCU CHEK Active, Roche Co.), were considered diabetic and selected for the study.

### Treatment of diabetes

Cinnamaldehyde was purchased from Sigma Aldrich and given to rats orally by gastric tube in a dose of 40 mg/kg/day for one month^19^. Metformin sold under the brand name Glucophage (a product of Merc Pharmaceuticals), was available in the form of tablets. Each tablet contains 1000 mg; these tablets were dissolved in distilled water and given to rats orally by gastric tube in a dose of 200 mg/kg/day for one month^20^.

### Experimental design

Animals were classified into the following groups: Group I (Control group): consisted of 8 healthy male rats that had not received any type of treatment, Group II (Diabetic group): consisted of 32 diabetic male rats which were subdivided into four subgroups (8 rats each): Group IIA: Untreated diabetic rats which received placebo treatment (distilled water orally daily for four weeks), Group IIB: Diabetic rats that were orally treated with cinnamaldehyde in a dose of 40mg/kg daily for four weeks^19^, Group IIC: Diabetic rats that were orally treated with metformin in a dose of 200 mg/kg daily for four weeks^20^, Group IID: Diabetic rats that were orally treated with a combination of cinnamaldehyde and metformin daily for four weeks. All rats followed a regimen of moderate exercise that reproduced an active lifestyle.

### Collection of samples

At the end of the treatment period, rats in all studied groups were overnight fasting, blood samples were collected from the retro-orbital vein under deep anesthesia using isoflurane (inhalation 100%) then all rats were sacrificed by decapitation. Adipose tissue and hepatic tissue were dissected from all rats included in the studied groups. The serum samples were prepared by collecting the blood from the retro-orbital vein in anticoagulant-free tubes, followed by centrifugation at 3000 × g for 10 min. The serum samples were used for the determination of fasting blood glucose level, insulin, HOMA-IR, triglycerides (TG), total cholesterol (TC), high-density lipoprotein-cholesterol (HDL-C), low-density lipoprotein-cholesterol (LDL-C)), alanine aminotransferase (ALT) activity, aspartate aminotransferase (AST) activity, urea, creatinine and total antioxidant capacity (TAC) levels^21,22^. The obtained adipose and hepatic tissue samples were used to determine Malondialdehyde (MDA) content and gene expression of mTOR, Beclin-1, LC3-II, Atg 5, and miR30a.

### Serum parameters measurements

Serum insulin concentration was determined following the instructions of the Insulin rat ELISA kit (EMD Millipore, USA), absorbance was measured at 450 nm and the homeostasis model assessment index for insulin resistance (HOMA-IR) was then calculated using the following formula:^23^

$$ {\text{HOMA - IR}} = \frac{{{\text{Fasting}}\;{\text{insulin}}\left( {\left( {\upmu {\text{IU}}} \right)/{\text{mL}}} \right){\text{fasting}}\;{\text{glucose}}\left( {{\text{mg}}/{\text{dL}}} \right)}}{22.5 \times 18} $$

Serum TG, TC, and HDL–C levels were determined by the enzymatic colorimetric method using reagents obtained from BioMed Diagnostics INC (USA), absorbance was measured at 546 nm. Serum LDL-C was calculated from TG, TC, and HDL-C concentrations using the following equation:^24^

$$ {\text{LDL}} - {\text{C}}\left( {{\text{mg}}/{\text{dL}}} \right) = {\text{TC}}{-}\left( {{\text{HDL}} - C} \right){-}{\text{TG}}/5 $$

Serum ALT and AST activities were determined using reagents obtained from BioMed Diagnostics INC (USA), absorbance was measured at 340 nm^25^. Urea and Creatinine were determined using reagents obtained from BioMed Diagnostics INC (USA), absorbance was measured at 570 nm and 510 nm, respectively^26,27^.

The Ferric reducing antioxidant power (FRAP) is a measure of TAC. The method described measures FRAP at low pH when a ferric-tripyridyltriazine Fe (III) -TPTZ complex is reduced to ferrous Fe (II) form, an intense blue color with an absorption maximum at 593 nm develops^28^. The total protein concentration was determined using Lowry’s method^29^.

### Determination of malondialdehyde (MDA) content as thiobarbituric acid reactive substances (TBARS)

Malondialdehyde was determined according to the method of Draper and Hadley. The tissue samples are heated with thiobarbituric acid (TBA) at low pH. The resulting pink chromogen has a maximal absorbance of 532 nm^30^.

### Gene expression analysis

Thirty mg of adipocyte and hepatic tissues were used for total RNA extraction using the miRNeasy Mini Kit (Qiagen, Germany) according to the manufacturer’s instructions, and the concentration and integrity of extracted RNA were checked using nanodrop. The reverse transcription of the extracted RNA was performed using Reverse transcription (RT) was performed by TOPscript™ RT DryMIX kit (dT18/dN6 plus) (Enzynomics, Korea) according to the manufacturer's instructions. The tissues expression of Beclin-1, LC3II, ATG5, mTOR, and miR 30a were quantified in the cDNA by CFX Maestro™ Software (Bio-Rad, USA) using QuantiNova™ SYBR® Green PCR Kit (Qiagen, Germany). Quantitative PCR amplification conditions were adjusted as an initial denaturation at 95 °C for 10 min and then 45 cycles of PCR for amplification as follows: Denaturation at 95 °C for 20 s, annealing at 55 °C for 20 s, and extension at 70 °C for 15 s. The housekeeping gene GADPH was used as a reference gene for normalization and U6 was used as a reference gene for miRNAs. The primers used for the determination of rat genes are presented in Table 1. The relative change in mRNA expression in samples was estimated using the 2-ΔΔCt method^31^.

**Table 1:** Primers sequence for real time-PCR.

Gene Name	Accession number of genes	Primer sequence	
mTOR	NM_019906.2	Forward	5′-TTGGAGTGGCTGGGTGCTGA-3′
		Reverse	5′-AAGGGCTGAACTTGCTGGAA-3′
Beclin-1	NM_001034117.1	Forward	5′-TTGGCCAATAAGATGGGTCTGAA-3′
		Reverse	5′-GTCAGGGACTCCAGATACGAGTG-3′
LC3-II	NM_022867.2	Forward	5′-CAGGATCCATGCCGTCCCAGAAGACC-3′
		Reverse	5′-GTCCCTTTTTGCCTTGGTAG-3′
Atg5	NM_001014250.1	Forward	5′-AACTGAAAGAGAAGCAGAACCA -3′
		Reverse	5′-TGTCTCATAACCTTCTGAAAGTGC-3′
GADPH	NR_046237.2	Forward	5′-AGTTAATGCCGCCCCTTACC-3′
		Reverse	5′- CAGGGCTGACTACAAACCCA-3′

### Statistical analysis

Data were analyzed using SPSS software package version 20.0 (SPSS Chicago, IL, USA). The data were expressed as mean ± SD. One-way analysis of variance (ANOVA) followed by post hoc Tukey test was used to compare the mean values between and within treated groups compared to untreated and control groups. Differences were considered statistically significant at P-value < 0.05. Correlation studies were performed using Pearson's correlation coefficient^32^.

## Results

### Parameters of glucose homeostasis

The untreated diabetic rats have marked significantly higher FBG and HOMA-IR levels but significantly lower insulin levels compared with control rats. All diabetic-treated rats showed significantly lower FBG and HOMA-IR but significantly higher insulin levels than untreated rats. The diabetic rats treated with a combination of cinnamaldehyde and metformin revealed significantly lower levels of FBG and significantly higher insulin levels compared with those treated with metformin or cinnamaldehyde alone (Table 2).

**Table 2:** The changes in serum parameter levels in the different studied groups.

Parameter	Control	Diabetic Rats			
		Untreated	Metformin treated	Cinnamaldehyde treated	Combination treated
FBG (mg/dl)	84.0 ± 9.65	335.0* ± 26.59	133.1*^a^ ± 2.80	149.3*^ab^ ± 2.76	121.9*^abc^ ± 7.0
Insulin (µIU/ml)	6.47 ± 0.42	4.79* ± 0.44	7.94*^a^ ± 0.45	7.86*^a^ ± 0.42	8.13*^abc^ ± 0.26
HOMA-IR	1.33 ± 0.18	3.98* ± 0.46	2.59*^a^ ± 0.15	2.88*^a^ ± 0.11	2.43*^a^ ± 0.12
TG (mg/dl)	83.08 ± 9.24	180.2* ± 5.59	110.3*^a^ ± 6.96	119.9*^ab^ ± 2.85	95.69*^abc^ ± 3.60
TC (mg/dl)	86.25 ± 4.80	110.3* ± 3.85	88.75^a^ ± 3.99	89.63^a^ ± 2.26	81.75*^abc^ ± 1.39
HDL–C (mg/dl)	59.88 ± 5.06	46.75* ± 2.96	54.63*^a^ ± 3.42	48.13*^a^ ± 4.58	53.0*^a^ ± 2.39
LDL-C (mg/dl)	9.89 ± 1.55	27.63* ± 5.90	12.10*^a^ ± 1.27	17.38*^a^ ± 5.43	9.56^abc^ ± 1.73
ALT(U/L)	35.0 ± 2.98	57.50* ± 5.10	47.38*^a^ ± 2.50	51.63*^ab^ ± 2.72	43.63*^abc^ ± 2.50
AST(U/L)	118.3 ± 11.83	172.6* ± 9.52	152.3*^a^ ± 3.33	163.4*^ab^ ± 2.13	140.6*^abc^ ± 1.85
Creatinine (mg/dl)	0.58 ± 0.07	0.78* ± 0.08	0.49*^a^ ± 0.06	0.56^ab^ ± 0.05	0.42*^abc^ ± 0.03
Urea (mg/dl)	53.63 ± 5.15	72.25* ± 5.82	57.38^a^ ± 2.92	59.13^a^ ± 9.14	52.75^ac^ ± 3.28
TAC (nmol/ml)	192.75 ± 9.07	168.75* ± 7.59	189.63^a^ ± 12.77	197.5^a^ ± 4.84	210.96*^abc^ ± 5.90

Comparison between groups was analyzed using one-way ANOVA followed by an LSD test. Data are presented as Mean ± SD.

*Significantly different from control rats.

^a^Significantly different from diabetic untreated rats.

^b^Significantly different from rats treated with metformin.

^c^Significantly different from rats treated with cinnamaldehyde.

### Serum of lipid profile

The levels of TG, TC, and LDL-C were significantly higher while HDL-C was significantly lower in the untreated diabetic rats compared with the healthy control group. The diabetic rats treated with cinnamaldehyde, metformin, or a combination of both showed significantly lower TG, TC, and LDL-C and significantly higher HDL-C levels compared with the untreated group. Diabetic rats treated with a combination of cinnamaldehyde and metformin have significantly lower TG, TC, and LDL-C than rats treated with metformin or cinnamaldehyde alone (Table 2).

The diabetic group treated with metformin or cinnamaldehyde showed no change in serum TC compared with control rats which means normalization of TC but significantly lower HDL-C level compared with control rats and significantly higher TG, TC, and LDL-C compared with the control group. The diabetic rats treated with a combination of cinnamaldehyde and metformin had no change in LDL-C level compared with the control group (Table 2).

### Liver function tests

The diabetic untreated rats have significantly higher ALT and AST levels compared with control rats. The diabetic rats treated with metformin have significantly lower ALT and AST levels than untreated diabetic rats but still higher than control rats. The diabetic rats treated with cinnamaldehyde have significantly lower ALT and AST levels than untreated diabetic rats but still higher than control rats and metformin-treated rats. The diabetic rats treated with a combination of metformin and cinnamaldehyde have significantly lower ALT and AST levels than untreated diabetic rats and other treated groups, but still higher than control rats (Table 2).

### Kidney function tests

The diabetic untreated rats have significantly higher serum creatinine and urea levels compared with control rats. All treated diabetic rats have significantly lower serum creatinine and urea levels than untreated diabetic rats. The diabetic rats treated with cinnamaldehyde alone or combined with metformin have significantly lower serum creatinine than metformin-treated rats. The diabetic rats treated with a combination of metformin and cinnamaldehyde have significantly lower serum creatinine and urea levels than cinnamaldehyde-treated rats. All diabetic-treated rats have no significant difference in serum urea level compared to control rats**.** Cinnamaldehyde-treated rats have no significant difference in serum creatinine levels compared to control rats, however, other treated groups showed significantly lower serum creatinine levels as compared to control (Table 2).

### Total antioxidant capacity (TAC)

The diabetic untreated rats have significantly lower TAC levels than control rats. The diabetic rats treated with metformin have significantly higher TAC levels compared with untreated diabetic rats with no significant difference compared with control rats. The diabetic rats treated with cinnamaldehyde have significantly higher TAC levels compared with untreated diabetic rats with no significant difference compared with control rats. The diabetic rats treated with a combination of metformin and cinnamaldehyde have significantly higher TAC levels compared with untreated diabetic rats, control rats, and all other treated groups (Table 2).

### Malondialdehyde (MDA) content

In both adipocyte and hepatic tissues, the diabetic untreated rats have significantly higher MDA content compared with control rats. The diabetic rats treated with metformin have significantly lower MDA content than untreated diabetic rats with no significant difference compared with control rats. The diabetic rats treated with cinnamaldehyde have significantly lower MDA content than untreated diabetic rats but still higher than control rats and diabetic rats treated with metformin. The diabetic rats treated with a combination of metformin and cinnamaldehyde have significantly lower MDA content than untreated diabetic rats and those treated with cinnamaldehyde alone (Table 3).

**Table 3:** The changes in MDA content in both adipocyte and hepatic tissues in the different studied groups.

Parameter	Control	Diabetic Rats			
		Untreated	Metformin treated	Cinnamaldehyde treated	Combination treated
Adipocyte MDA (nmol/gm tissue)	3.71 ± 0.49	6.79* ± 0.58	3.43^a^ ± 0.42	4.58*^ab^ ± 0.69	3.06^ac^ ± 0.47
Hepatic MDA (nmol/gm tissue)	3.70 ± 0.45	6.69* ± 0.55	3.45^a^ ± 0.42	4.36^ab^ ± 0.76	2.99*^abc^ ± 0.41

Comparison between groups was analyzed using one-way ANOVA followed by an LSD test. Data are presented as Mean ± SD.

*Significantly different from control rats.

^a^Significantly different from diabetic untreated rats.

^b^Significantly different from rats treated with metformin.

^c^Significantly different from rats treated with cinnamaldehyde.

### mTOR gene expression

**Figure 1:**
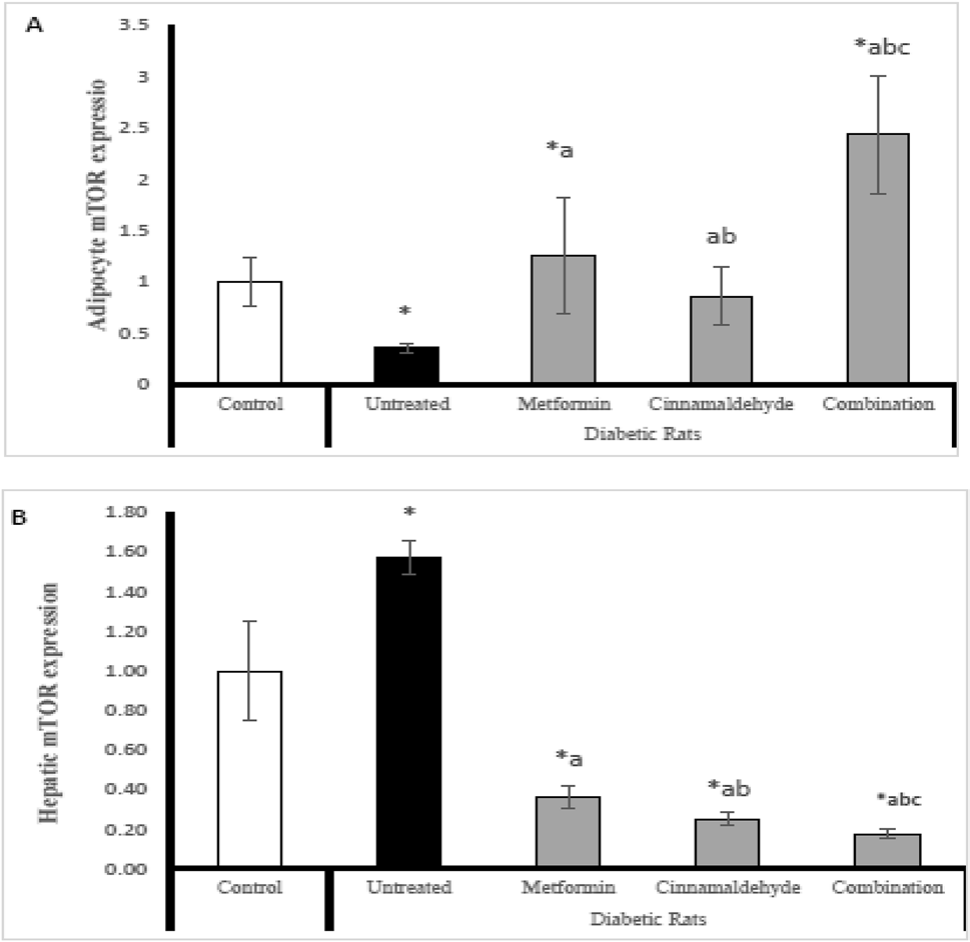
(**A**) Adipocyte mTOR expression, (**B**) hepatic mTOR expression in the different studied groups. Comparison between groups was analyzed using one-way ANOVA followed by an LSD test. Data are presented as Mean ± SD,*: significantly different from control rats. a: significantly different from diabetic untreated rats. b: significantly different from rats treated with metformin.c: significantly different from rats treated with cinnamaldehyde.

In adipocyte; The untreated diabetic rats have significantly lower adipose tissue expression of mTOR compared with control rats. The diabetic rats treated with metformin have significantly higher expression of mTOR than untreated diabetic rats and control rats. The diabetic rats treated with cinnamaldehyde have significantly higher expression of mTOR than untreated diabetic rats and lower than metformin-treated rats. The diabetic rats treated with a combination of cinnamaldehyde and metformin have significantly higher mTOR expression compared with control rats, untreated diabetic rats, and all other treated groups (Fig. 1A).

However in hepatic tissue; The untreated diabetic rats have a significantly higher hepatic expression of mTOR compared with control rats and all other treated groups. The diabetic rats treated with metformin have significantly lower expression of mTOR than untreated diabetic rats and control rats. The diabetic rats treated with cinnamaldehyde have significantly lower expression of mTOR than the untreated group, metformin-treated group and control group. The diabetic rats treated with a combination of metformin and cinnamaldehyde have significantly lower expression of mTOR compared with control rats, untreated diabetic rats, and all other treated groups (Fig. 1B).

### Beclin-1 gene expression

**Figure 2:**
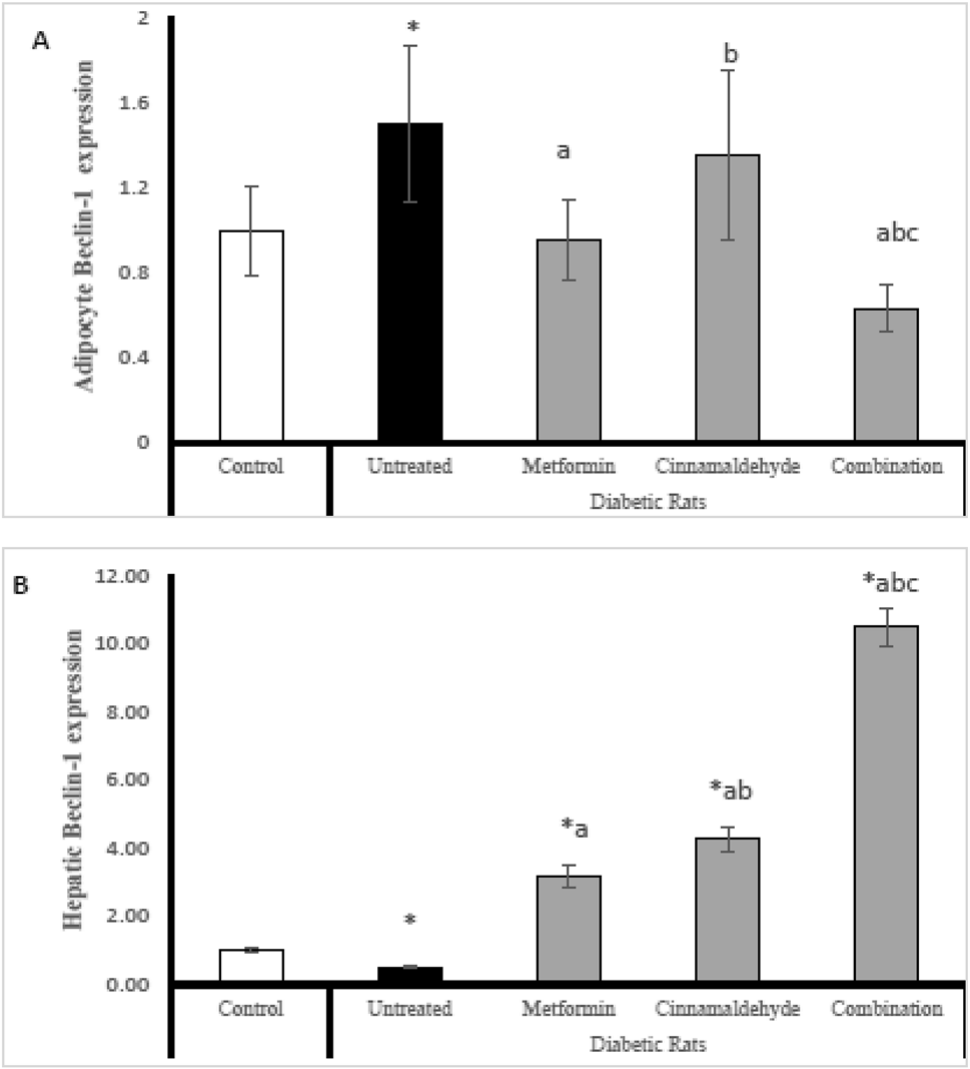
(**A**) Adipocyte Beclin-1 expression, (**B**) hepatic Beclin-1 expression in the different studied groups. Comparison between groups was analyzed using one-way ANOVA followed by an LSD test. Data are presented as Mean ± SD,*: significantly different from control rats. a: significantly different from diabetic untreated rats. b: significantly different from rats treated with metformin. c: significantly different from rats treated with cinnamaldehyde.

In adipocyte; The untreated diabetic rats have significantly high adipose tissue expression of Beclin-1 compared with control rats. The diabetic rats treated with metformin have significantly lower Beclin-1 expression than untreated diabetic rats. The diabetic rats treated with cinnamaldehyde have significantly higher Beclin-1 expression than metformin-treated rats. The diabetic rats treated with a combination of cinnamaldehyde and metformin have significantly higher expression of Beclin-1 compared with untreated diabetic rats and all other treated groups (Fig. 2A).

However in hepatic tissues; The diabetic untreated rats have significantly lower expression of Beclin-1 compared with control rats. The diabetic rats treated with metformin have significantly higher expression of Beclin-1 than untreated diabetic rats and control rats. The diabetic rats treated with cinnamaldehyde have significantly higher expression of Beclin-1 than the untreated group, metformin-treated group and control group. The diabetic rats treated with a combination of metformin and cinnamaldehyde have significantly higher expression of Beclin-1 compared with control rats, untreated diabetic rats, and all other treated groups (Fig. 2B).

### LC3-II gene expression

**Figure 3:**
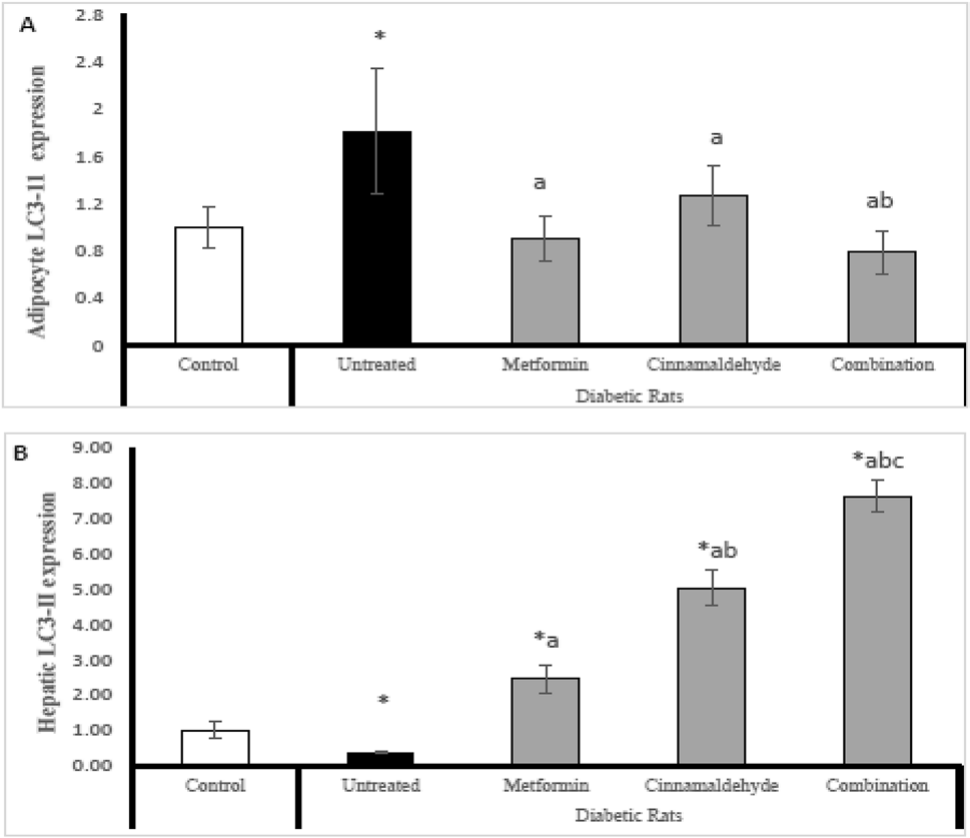
(**A**) Adipocyte LC3-II expression, (**B**) hepatic LC3-II expression in the different studied groups. Comparison between groups was analyzed using one-way ANOVA followed by an LSD test. Data are presented as Mean ± SD,*: significantly different from control rats. a: significantly different from diabetic untreated rats. b: significantly different from rats treated with metformin. c: significantly different from rats treated with cinnamaldehyde.

In adipocyte; The untreated diabetic rats have significantly high adipose tissue expression of LC3-II compared with control rats. All the diabetic-treated rats have significantly low expression of LC3-II compared with untreated diabetic rats with no significant change with the control group. The diabetic rats treated with a combination of metformin and cinnamaldehyde have significantly lower expression of LC3-II compared with metformin-treated groups (Fig. 3A).

However in hepatic tissues; The diabetic untreated rats have significantly lower expression of LC3-II compared with control rats. The diabetic rats treated with metformin have significantly higher expression of LC3-II than untreated diabetic rats and control rats. The diabetic rats treated with cinnamaldehyde have significantly higher expression of LC3-II than the untreated group, metformin-treated group, and control group. The diabetic rats treated with a combination of metformin and cinnamaldehyde have significantly higher expression of LC3-II compared with control rats, untreated diabetic rats, and all other treated groups (Fig. 3B).

### Atg5 gene expression

**Figure 4:**
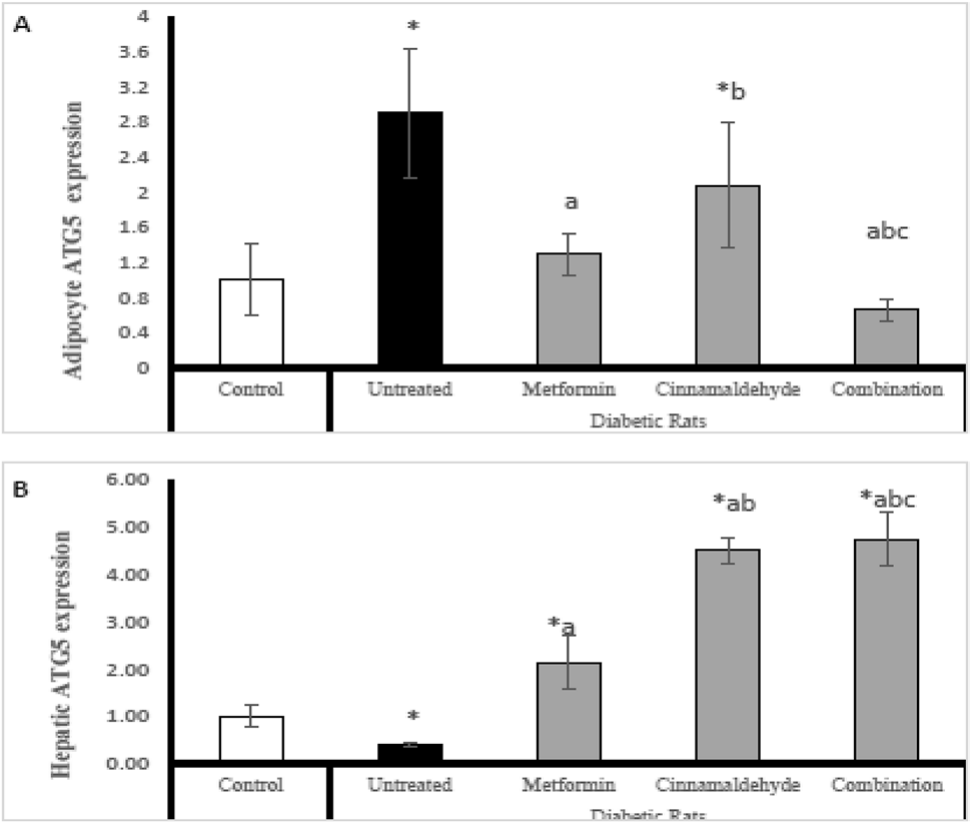
(**A**) Adipocyte Atg5 expression, (**B**) hepatic Atg5 expression in the different studied groups. Comparison between groups was analyzed using one-way ANOVA followed by an LSD test. Data are presented as Mean ± SD,*: significantly different from control rats. a: significantly different from diabetic untreated rats. b: significantly different from rats treated with metformin. c: significantly different from rats treated with cinnamaldehyde.

In adipocytes; The untreated diabetic rat's adipose tissue has significantly higher expression of adipose tissue of Atg5 expression compared with control rats. The diabetic rats treated with metformin have significantly lower expression of Atg5 compared with untreated diabetic rats. The diabetic rats treated with cinnamaldehyde have significantly higher expression of Atg5 compared with metformin-treated rats and the control group. The diabetic rats treated with metformin and a combination of cinnamaldehyde and metformin have significantly lower Atg5 expression than untreated and other treated groups but have no significant difference from the control group (Fig. 4A).

However in hepatic tissues; The diabetic untreated rats have significantly lower expression of Atg5 compared with control rats. The diabetic rats treated with metformin have significantly higher expression of Atg5 than untreated diabetic rats and control rats. The diabetic rats treated with cinnamaldehyde have significantly higher expression of Atg5 than the untreated group, metformin-treated group and control group. The diabetic rats treated with a combination of metformin and cinnamaldehyde have significantly higher expression of Atg5 compared with control rats, untreated diabetic rats, and all other treated groups (Fig. 4B).

### miR30a expression

**Figure 5:**
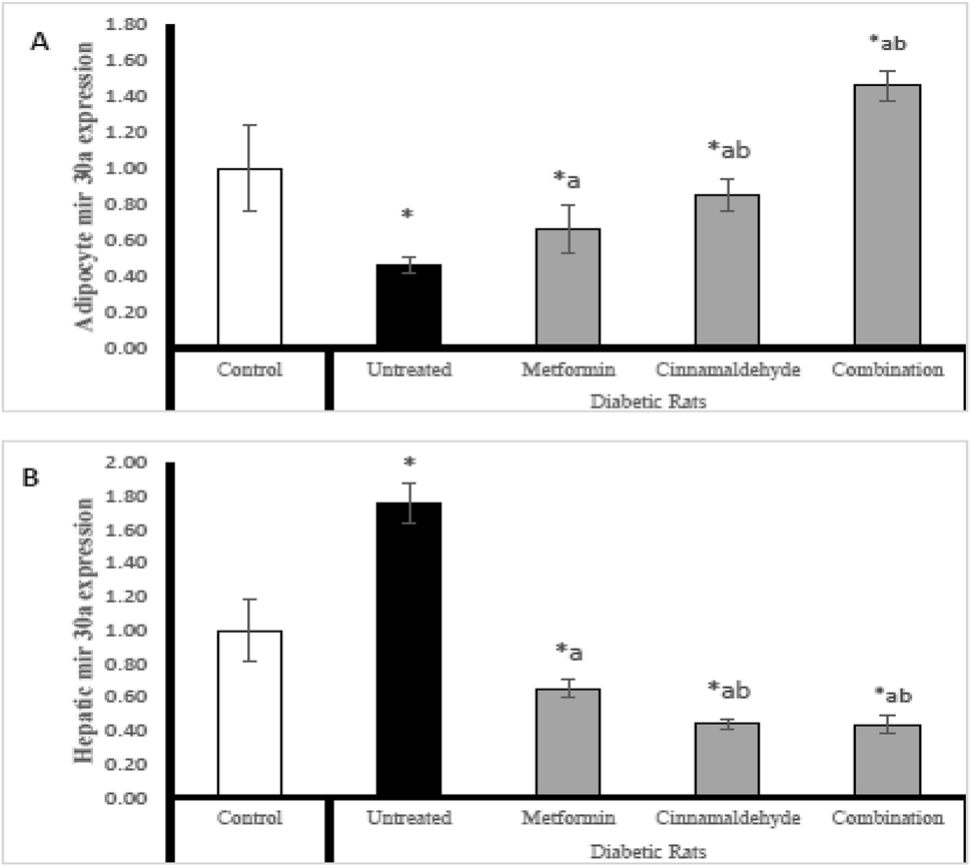
(**A**) Adipocyte mir 30a expression, (**B**) hepatic mir 30a expression in the different studied groups. Comparison between groups was analyzed using one-way ANOVA followed by an LSD test. Data are presented as Mean ± SD,*: significantly different from control rats. a: significantly different from diabetic untreated rats.b: significantly different from rats treated with metformin. c: significantly different from rats treated with cinnamaldehyde.

In adipocyte; The diabetic untreated rats have significantly lower expression of mir30a compared with control rats. The diabetic rats treated with metformin have significantly higher expression of mir30a than untreated diabetic rats but lower than control rats. The diabetic rats treated with cinnamaldehyde have significantly higher expression of mir30a than the untreated group, and metformin-treated group but lower than the control group. The diabetic rats treated with a combination of metformin and cinnamaldehyde have significantly higher expression of mir30a compared with untreated diabetic rats, control rats, and metformin-treated rats (Fig. 5A).

However in hepatic tissues; The diabetic untreated rats have significantly higher expression of mir30a compared with control rats. The diabetic rats treated with metformin have significantly lower expression of mir30a than untreated diabetic rats and control rats. The diabetic rats treated with cinnamaldehyde alone or combined with metformin have significantly lower expression of mir30a than the untreated group, metformin-treated group, and control group (Fig. 5B).

### Adipocyte correlation studies

**Figure 6:**
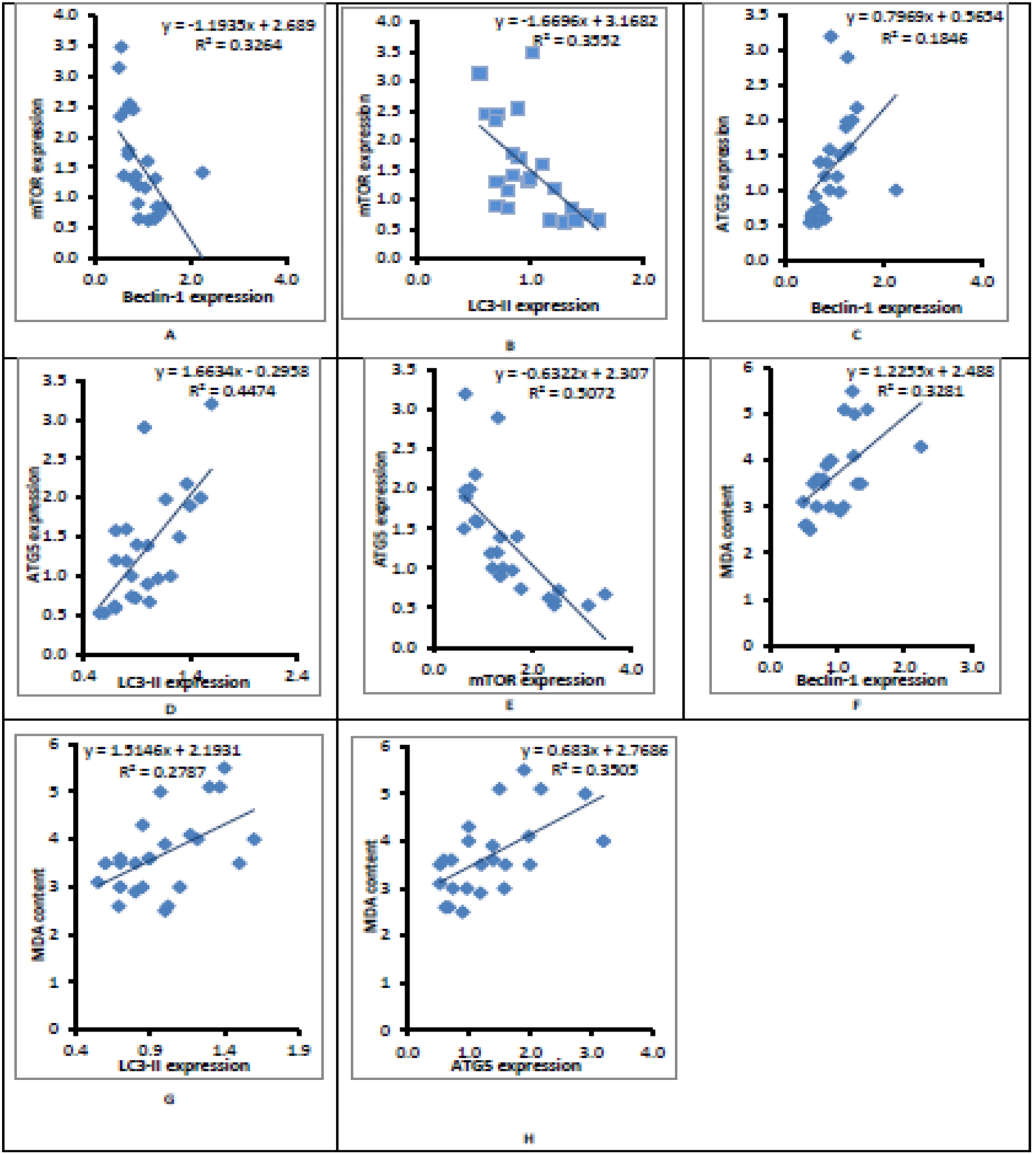
Adipocyte correlation studies.

- Adipocyte mTOR was negatively correlated with Beclin-1 expression (*r* = − 0.571, *p* = 0.004) (Fig. 6A) and LC3-II expresion (*r* = − 0.596, *p* = 0.002) (Fig. 6B).
- Adipose tissue Atg5 was positively correlated with Beclin-1 expression (*r* = 0.430, *p* = 0.036) (Fig. 6C) and LC3-II expresion (*r* = 0.669, *p* < 0.001) (Fig. 6D) while negatively correlated with mTOR expression (*r* = − 0.712, *p* < 0.001) (Fig. 6E).
- Adipocyte MDA was positively correlated with Beclin-1 expression (*r* = 0.573, *p* < 0.003) (Fig. 6F) and LC3-II expresion (*r* = 0.528, *p* = 0.008) (Fig. 6G) and Atg5 expresion (*r* = 0.592, *p* = 0.002) (Fig. 6H).

### Hepatic correlation studies

**Figure 7:**
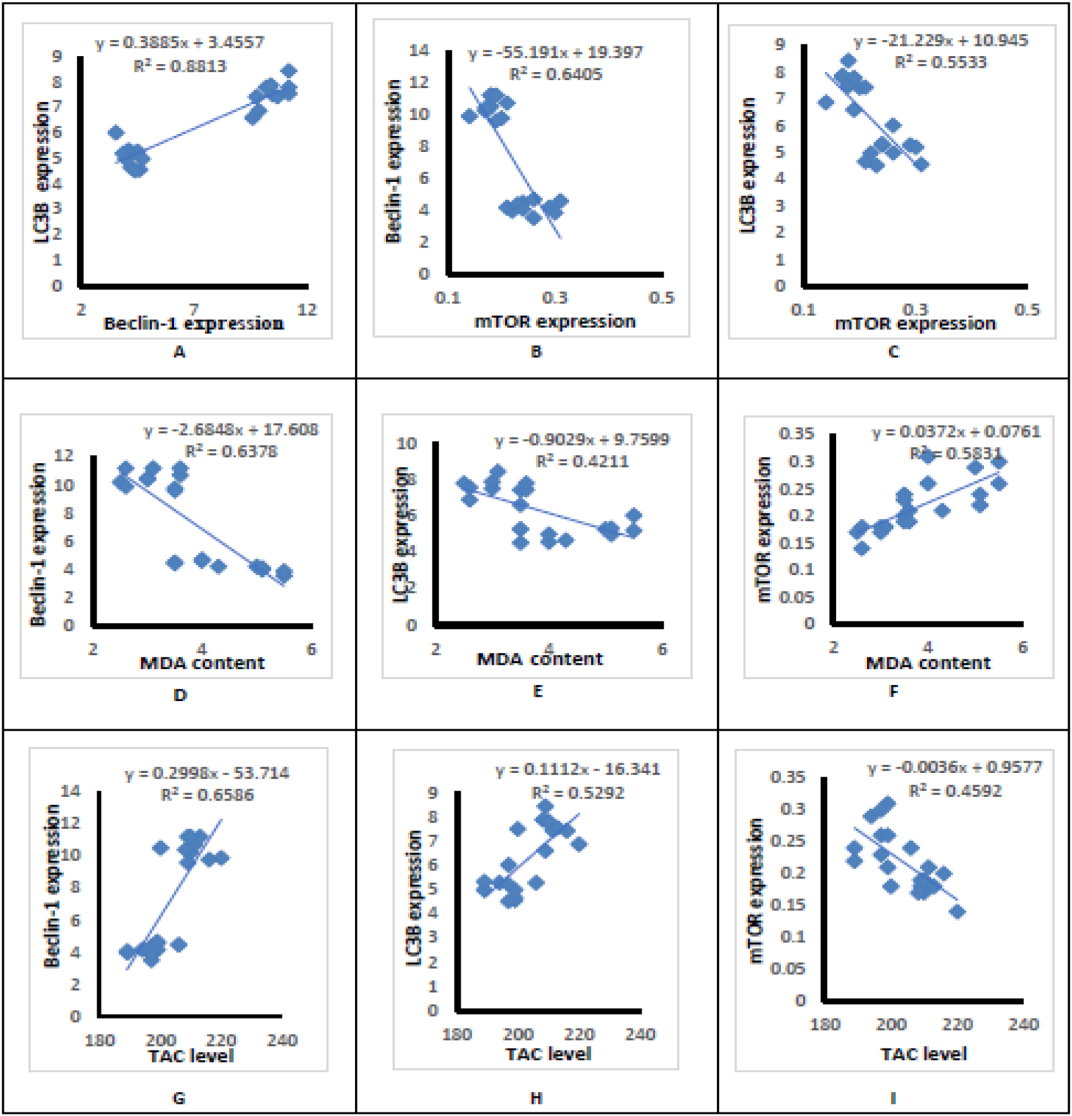
Hepatic correlation studies.

- Hepatic Beclin-1 expression was positively correlated with LC3-II (*r* = 0.939, *p* < 0.001) (Fig. 7A).
- Hepatic mTOR expression was negatively correlated with Beclin-1 expression (*r* = **− **0.802, *p* < 0.001) (Fig. 7B) and LC3-II expression (*r* = − 0.745, *p* < 0.001) (Fig. 7C).
- Hepatic MDA content was negatively correlated with Beclin-1 expression (*r* = − 0.799, *p* < 0.001) (Fig. 7D) and LC3-II expression (*r* = − 0.645, *p* < 0.001) (Fig. 7E) and positively correlated with mTOR expression (*r* = 0.767, *p* < 0.001) (Fig. 7F).
- Hepatic TAC level was positively correlated with Beclin-1 expression (*r* = 0.812, *p* < 0.001) (Fig. 7G) and LC3-II expression (*r* = − 0.727, *p* < 0.001) (Fig. 7H) and negatively correlated with mTOR expression (*r* = − 0.680, *p* < 0.001) (Fig. 7I).

## Discussion

The autophagic machinery plays a role in the pathogenesis of T2DM and controls the normal pancreatic beta cell activity. This study came to investigate the different therapeutic effects of cinnamaldehyde on adipocyte and hepatic autophagy in the High-fat diet/ Streptozotocin-diabetic rats´ model compared to the traditional drug metformin. In the current study, an HFD/STZ rat model was used as an animal model of T2DM. The modified Srinivasan et al. approach was used to induce diabetes; HFD was followed by a single low dose of STZ given intraperitoneally at a rate of 55 mg/kg of body weight^17^. While STZ at low doses mildly impairs pancreatic β-cell function, HFD develops insulin resistance^18^. According to the current study, the HFD/STZ diabetic rats showed overt hyperglycemia, dyslipidemia, hypoinsulinemia, and insulin resistance, which is shown by an increase in HOMA-IR. This type 2 diabetic rat model exhibited characteristics that closely resemble the metabolic characteristics of T2DM in people, making it appropriate for pharmacological research.

The anomalies in the lipid profile exhibiting a significantly elevated TG, TC, and LDL-C as well as a significantly decreased HDL-C, which are collectively known as diabetic dyslipidemia, were linked to the disrupted glucose homeostasis in the HFD/STZ diabetic rat model. These abnormalities may result from or be a cause of diabetes and insulin resistance^33^. Although the preventive management of these problems reduces the likelihood of dyslipidemia, it is still considered one of the main risk factors for cardiovascular disease in T2DM, which has a high mortality and morbidity rate^34^. The hepatic enzymes AST and ALT were raised along with the dyslipidemia seen in the diabetic rats, indicating hepatocyte inflammation that may have been caused by fat buildup and the induction of fatty liver as a hepatic manifestation of metabolic disturbances in HFD/STZ-diabetic rats^35^. The current results clarified that the untreated diabetic rats had increased ALT, AST, urea, and creatinine levels, which may be due to the oxidative stress that was indicated by the increased MDA level and the decreased TAC level. To maintain nutritional balance and enforce quality control within the cell, autophagy, which is a conserved biological mechanism, recycles damaged organelles and long-lived proteins^36^.

When compared to control rats in the study, the HFD/STZ diabetic rats displayed different expressions of the mTOR gene where there is a significant up-regulation in hepatic tissues while a significant down-regulation in adipocytes. Given the importance of metabolic control and mTOR's role as a primary sensor and regulator of cellular energy, it is not surprising that this signaling is dysregulated in a variety of disease states like cancer and type 2 diabetes^37,38^ In the liver, muscle, and pancreatic islets of obese and high-fat-fed mice, mTOR activity is noticeably raised; this is associated to elevated pro-inflammatory cytokines, and nutrients like glucose^39^. Saxton and Sabatini, and found that autophagy in adipose tissues of T2D and obese rats have markedly downregulated mTOR protein expression. Multicellular organisms must develop essential mechanisms to detect and adjust to the constantly varying extracellular environment to survive and grow^40^.

Autophagy is negatively regulated by mTOR^41^. Activation of mTOR complexes inhibits autophagy through the phosphorylation of many autophagy-related proteins, like ULK1, Atg13,& Atg14L. These proteins are essential to initiate autophagy and autophagosome formation^42^.

In HFD-STZ-induced type 2 diabetes, Beclin-1 levels were considerably upregulated in the adipose tissue of diabetic rats than in control tissues, indicating the start of autophagosome formation and inducing the cellular autophagic process. This result is in alignment with that of Sun et al., who discovered that Beclin-1 gene expression levels were much greater in the cells of rats fed with HFD than in the cells of rats fed a regular diet as controls^43^.

Increased autophagy was reported in the adipose tissue of patients with diabetes and obesity. In adipocytes from visceral and subcutaneous fat depots of obese and T2D individuals, the level of the autophagy marker microtubule-associated protein light chain3 (LC3), a protein that was predominantly detected in cells of the adipose tissue, was shown to be higher. In visceral adipose tissue of non-diabetic obese and T2D patients as compared to lean individuals, up-regulated expression of LC3-II form was found indicating that autophagy was upregulated in adipose tissue of T2D patients^44^.

Regarding The expression of the Atg 5, which is necessary for the lysosomal breakdown of organelles and proteins, which is continuously destroyed during autophagy. Compared to non-diabetic, lean people, T2D and obese patients have higher levels of Atg5 protein expression in their adipose tissue. Moreover, visceral AT of T2D and obese patients have a significant increase in LC3 and Atg5 gene expression, suggesting an active autophagic process in these patients^45^. In line with these observations results of the present study retrieved significant upregulation in the expression of LC3-II, Atg5, and Beclin-1 in adipose tissue of HFD/STZ diabetic rats compared with nondiabetic controls suggesting an active autophagic process in type 2 diabetes.

However, the results of this study showed an inversed pattern of hepatic autophagy, it showed down-regulation of hepatic expression of Beclin-1, LC3II, and Atg5 in HFD/STZ diabetic rats than control rats. Activation of Beclin-1 by phosphorylation increases VPS34 kinase activity, leading to an increase in autophagy^46,47^.

This study revealed that diabetic rats had higher hepatic expression of miR-30a and lower expression in adipocytes compared with normal rats. On human chromosome 6q, miR-30a, a member of the miR-30 family, is located^48^. Recent research has demonstrated that miR-30a reduces Beclin-1 activity, preventing the formation of autophagic vesicles and the beginning of autophagy^49^.

The current investigation supports metformin's anti-diabetic effects in diabetic rats with the HFD/STZ model, as it significantly declines the fasting blood sugar, HOMA-IR, TC, and TG. The most often prescribed medication for type 2 diabetes globally is metformin, a biguanide, which can be used alone or in combination with other glucose-lowering treatments such as insulin^50^.

In our study, the presented data demonstrated activation of autophagy in adipose tissue and reduction in hepatic tissues of diabetic rats treated with HFD/STZ and modulation after treatment with metformin. This was manifested by up-regulation of mTOR gene expression and downregulation of Beclin-1, LC3-II, and Atg 5 gene expression after treatment with metformin in adipose tissue. In the adipose tissues of the HFD rats, metformin reduced the induction of autophagy while directly enhancing the induction of hepatic autophagy^51^. So it was observed upregulation in mTOR gene expression and downregulation of LC3-II, also Lettieri Barbato's study showed with the inhibition of autophagy in adipose tissue a significant drop in gene expression of Beclin 1 and Atg 5^52^.

There aren't many clinical studies on metformin's effects on autophagy in diabetes, but those that do suggest that autophagy may be a key target of metformin playing pharmacological effects. Diabetes is the most common disease connecting autophagy in the application of metformin. Numerous investigations revealed that the key molecule in metformin's pharmacological activities is AMPK. Further research revealed that AMPK can control the autophagy process by controlling several downstream signaling molecules, including mTOR and ULK1^53,54^.

Activated AMPK can inhibit the crucial downstream molecule mTOR, which then has comparable consequences. mTOR inhibition enhanced the interaction between AMPK and ULK1. Another essential AMPK downstream signaling molecule involved in activating autophagy is ULK1. Macroautophagy can be brought on by AMPK's activation of ULK1^55^. Beclin-1 is phosphorylated on Ser 14 as a result of ULK1 activation, which also increases the activity of VPS34 complexes that contain Atg14L. Mammals require the Ser 14 phosphorylation of Beclin-1 by ULK to fully induce autophagy^56^. ULK1, the joint target of AMPK and suppressed mTOR, is therefore essential for autophagy (especially in severe nutrient deprivation). Further research was necessary, nevertheless, to determine how metformin's interactions with AMPK/ULK1 and AMPK/mTOR affect autophagy.

Kim et al., revealed that metformin can induce autophagy in hepatic tissues through the downregulation of miRNA expression^57^. Metformin removes the inhibitory effect of miR30a on Beclin-1 leading to the induction of autophagy, also Liu, Yue^58^ reported that metformin induces the protein expression of Beclin-1 and the accumulation of LC3 II resulting in the induction of autophagy. As mentioned before, miR30a downregulates the gene expression of Beclin-1 resulting in the inhibition of the autophagic pathway^59^.

Results of the present study indicated that cinnamaldehyde normalized the fasting blood glucose in diabetic rats and decreased HOMA-IR. This finding is by Çelik, Mert^60^ and Sierra-Puente, Abadi-Alfie^61^ who reported that the cinnamaldehyde reduced the blood glucose levels. A proposed mechanism of FBG lowering effect of cinnamaldehyde was mentioned in Anand, Murali^62^ study who reported the ability of cinnamaldehyde to activate the phosphoenol pyruvate carboxy kinase (PEPCK) and pyruvate kinase (PK), which promote the synthesis of glycogen and the inhibition of gluconeogenesis.

In the present study, the diabetic rats treated with cinnamaldehyde have significantly higher insulin levels compared to untreated rats. In addition, they have lower TG and TC levels than untreated rats. These results are in line with Sharma, Mandal^63^ and Kumar, Vasudeva^64^ who reported an increase in insulin levels in diabetic rats treated with cinnamaldehyde and a decrease in the level of serum triglycerides (TGs) and total cholesterol (TC). The inhibition of adipose tissue lipolysis is the most potent effect of insulin. Insulin affects the mobilization of fatty acids from adipose tissue. While a fall in basal insulin levels causes a noticeable acceleration of lipolysis, a rise in plasma insulin concentration of just 5 IU/ml suppresses it by 50%. Increased insulin levels may limit lipolysis and lower plasma triglyceride and cholesterol levels^64^. This mechanistic explanation could clarify the cinnamaldehyde effect on TGs and TC in diabetic rats in the current study.

A study by Subash-Babu, Alshatwi^65^ reported that cinnamaldehyde significantly increased the amount of insulin released, most likely by preventing free radical damage to β-cells through its antioxidant action.

In diabetic rats treated with cinnamaldehyde, the oxidative stress decreased, and this was confirmed by decreased MDA level and increased TAC level. As a result, the levels of ALT, AST, urea, and creatinine decreased. These findings are in agreement with Rashwan, El-Beltagy^66^ who reported that the cinnamaldehyde decreased the level of ALT, AST, creatinine, and urea.

Knowledgeably, there are not enough studies that demonstrate the cinnamaldehyde effect on hepatic and adipocyte autophagy in the HFD/STZ diabetic rat model. Surprisingly, cinnamaldehyde treatment reduced adipocyte autophagy and induced hepatic autophagy in diabetic rats, which is confirmed by the change expression of autophagic markers: Beclin-1, LC3 II, and Atg5 and by the change in the expression of mTOR and miR30a compared with untreated diabetic rats. The exact molecular mechanism(s) concerned with the effect of cinnamaldehyde on autophagy in diabetes is unclear.

The present results proposed that induction of hepatic autophagy may be due to the downregulated expression of miR-30a that is correlated with the upregulated expression of Beclin-1 and LC3 II. This proposal was confirmed by the negative correlation between the level of Beclin-1 expression and miR-30a. miR-30a downregulates Beclin-1 expression, resulting in decreased autophagic activity^59,67^ and Chung, Kim^68^ reported that cinnamaldehyde treatment induces autophagy by increasing the expression of Beclin-1 and LC3 II. Furthermore, the downregulated mTOR expression leads to ULK1 dephosphorylation and induction of autophagy^69^. The activated (dephosphorylated) ULK1 relocates Beclin-1 to ER and phosphorylates mAtg13 and FIP200 initiating autophagy^70^. The present investigations illustrated that the effect of cinnamaldehyde on the autophagic markers, Beclin-1, LC3II, mTOR, and miR30a was potentiated when it was used in combination with metformin.

In addition, we present further evidence that increased markers of adipose tissue autophagy reflect an increase in adipocyte autophagosomes. According to our research, obese type 2 diabetic (T2D) rats have been found to have adipose tissue-activated autophagy, which is characterized by increased expression of the autophagy genes Atg5, LC3-II, and Beclin -1 and decreased expression of mTOR. Additionally, cinnamaldehyde has been shown to reduce autophagy in the adipose tissue of HFD/STZ diabetic rats by increasing mTOR gene expression and decreasing gene expression of Beclin -1, Atg 5, or LC3-II whether used alone or in conjunction with metformin.

Combination therapy has emerged as one of the most successful treatments for treating type 2 diabetes, particularly in people who are more likely to develop problems linked to the disease^71^. This is crucial since a single treatment, such as the oral administration of metformin, may not be enough to support the body's ability to manage glucose levels over an extended length of time^72^.

Before autophagy might be targeted as a therapeutic approach, more research is required due to the complicated regulation of autophagy in obesity and diabetes. Given its obvious functions in normal physiology, it would seem that regulating autophagy may be utilized to treat individuals whose disease process has altered autophagic activity. This would aim to normalize autophagy rather than artificially manipulate it. These investigations will make it possible to identify diabetic patients who might respond favorably to medication that modulates autophagy. They will also give important guidance on when and how autophagy should be modulated in the process of treating patients with diabetes and/or obesity.

From previous discussion, it could be concluded that cinnamaldehyde, a naturally occurring agent could lower glucose levels and lipids, and treat diabetes by modulating autophagy in both adipose and hepatic tissues. It can also be used in conjunction with metformin to improve its ability to treat diabetes and minimize its side effects.

## Conclusions

**Figure 8:**
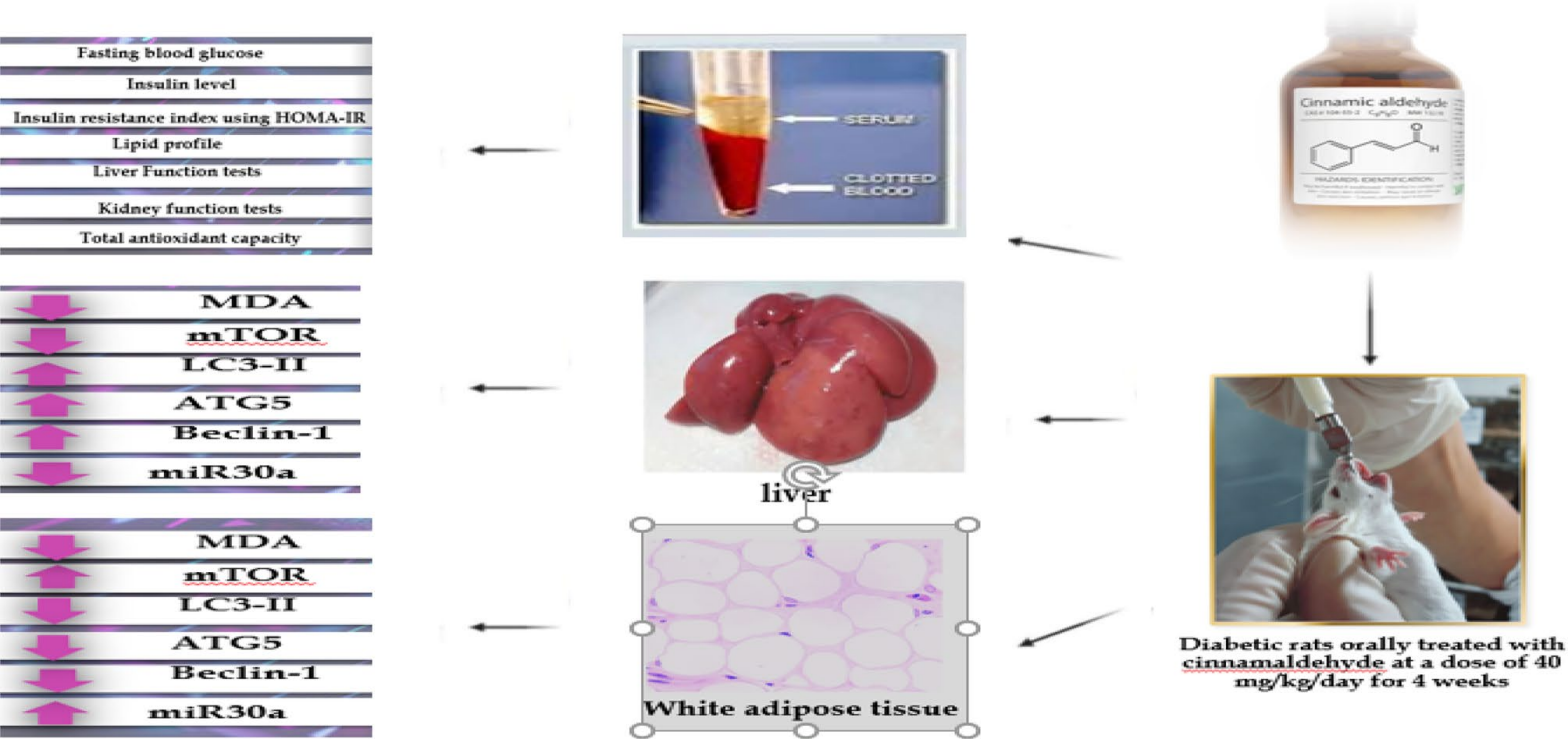
Graphical abstract.

From the results of the present study and the above discussion, it could be concluded that cinnamaldehyde is a promising natural glucose and lipid-lowering agent that has a vital role in the treatment of diabetes. In adipose tissues, It's interesting to note that cinnamaldehyde therapy decreased autophagy by upregulating mTOR, and mir30a gene expression and downregulating Beclin-1, LC3- II, and Atg5 gene expression. In hepatic tissues, Cinnamaldehyde treatment induces autophagy via upregulating the expression of autophagy markers, Beclin-1, LC3-II, and Atg5 and downregulating the expression of mTOR and miR30a. Also, decreases the oxidative stress which is indicated by the decreased MDA content in both tissues and the increased TAC serum level (Figure 8). After all, utilizing cinnamaldehyde in conjunction with metformin has demonstrated superior improvement in glucose homeostasis measures, lipid profile, and inflammatory, and autophagic gene expression. Thus, cinnamaldehyde could be used as an adjuvant therapy with metformin to boost the treatment efficacy and decrease the unwanted effect of metformin. Further studies are required to confirm these results by studying the change in autophagy at the protein levels. Future studies are required to explore the molecular mechanism(s), pharmacodynamics, and pharmacokinetics of cinnamaldehyde. The effect of lower doses of cinnamaldhyde needs to be explored.

## Data Availability

The data used and/or analyzed during the current study is available from the corresponding author upon reasonable request.
